# Effect of fluid shear stress on *in vitro* cultured ureteric bud cells

**DOI:** 10.1063/1.5035328

**Published:** 2018-07-10

**Authors:** Hiroshi Kimura, Masaki Nishikawa, Naomi Yanagawa, Hiroko Nakamura, Shunsuke Miyamoto, Morgan Hamon, Peter Hauser, Lifu Zhao, Oak D. Jo, Mitsuru Komeya, Takehiko Ogawa, Norimoto Yanagawa

**Affiliations:** 1 Department of Mechanical Engineering, School of Engineering, Tokai University, Hiratsuka, Kanagawa 259-1292, Japan; 2 Micro/Nano Technology Center, Tokai University, Hiratsuka, Kanagawa 259-1292, Japan; 3 Medical and Research Services, Greater Los Angeles Veterans Affairs Healthcare System at Sepulveda, North Hills, California 91343, USA; 4 Department of Medicine, University of California at Los Angeles, David Geffen School of Medicine, Los Angeles, California 90095, USA; 5 Department of Urology, Yokohama City University Graduate School of Medicine, Yokohama, Kanagawa 236-0004, Japan; 6 Institute of Molecular Medicine and Life Science, Yokohama City University Association of Medical Science, Yokohama, Kanagawa 236-0004, Japan

## Abstract

Most kidney cells are continuously exposed to fluid shear stress (FSS) from either blood flow or urine flow. Recent studies suggest that changes in FSS could contribute to the function and injury of these kidney cells. However, it is unclear whether FSS influences kidney development when urinary flow starts in the embryonic kidneys. In this study, we evaluated the influence of FSS on *in vitro* cultured ureteric bud (UB) cells by using a pumpless microfluidic device, which offers the convenience of conducting parallel cell culture experiments while also eliminating the need for cumbersome electronic driven equipment and intricate techniques. We first validated the function of the device by both mathematical model and experimental measurements. UB cells dissected from E15.5 mouse embryonic kidneys were cultured in the pumpless microfluidic device and subjected to FSS in the range of 0.4–0.6 dyn mm^−2^ for 48 h (dynamic). Control UB cells were similarly cultured in the device and maintained under a no-flow condition (static). We found from our present study that the exposure to FSS for up to 48 h led to an increase in mRNA expression levels of UB tip cell marker genes (*Wnt11*, *Ret*, *Etv4*) with a decrease in stalk cell marker genes (*Wnt7b*, *Tacstd2*). In further support of the enrichment of UB tip cell population in response to FSS, we also found that exposure to FSS led to a remarkable reduction in the binding of lectin Dolichos Biflorus Agglutinin. In conclusion, results of our present study show that exposure to FSS led to an enrichment in UB tip cell populations, which could contribute to the development and function of the embryonic kidney when urine flow starts at around embryonic age E15.5 in mouse. Since UB tip cells are known to be the proliferative progenitor cells that contribute to the branching morphogenesis of the collecting system in the kidney, our finding could imply an important link between the FSS from the initiation of urine flow and the development and function of the kidney.

## I. INTRODUCTION

It is well known that fluid shear stress (FSS) influences cell functions such as alignment, migration, differentiation, and phenotypic expression.^1^ In the kidney, there are two types of fluid flows, i.e., blood flow and urine flow. Therefore, it is conceivable that FSS may affect the functions of kidney cells and play a role in various kidney diseases.^2,3^

In recent years, microfluidic technology has been widely used to study the effect of FSS on kidney cells.^1,4–6^ These studies showed that exposure to FSS in the range of 0.2–5.0 dyn cm^−2^ that mimicks the *in vivo* condition had significant effects on not only kidney cell morphology, such as orientation, thickness, and cilia formation, but also kidney cell functions, such as albumin transport, glucose reabsorption, and alkaline phosphatase activity.^7–10^ On the other hand, higher levels of FSS were found to cause marked reduction in cell viability and reduced levels of urokinase release.^11^

The kidney is a complex organ that consists of more than 20 different types of cells organized in a three-dimensional structure and plays a critical role in maintaining the homeostasis of our body.^12^ This complex organ, however, develops from a rather simple structure, called metanephros, which consists of mainly three lineages of progenitor cells derived from the intermediate mesoderm, i.e., metanephric mesenchymal (MM) cells, ureteric bud (UB) cells, and stromal (SM) cells. The development of the metanephros begins with the invasion of UB cells into MM cells at embryonic day 10.5 (E10.5) in mouse. Upon this UB invasion, condensed MM cell aggregates surround the tip of the invading UB, forming what is called the cap mesenchyme (CM), while SM cells create an outer layer covering the CM.^13,14^ Thereafter, mutual interactions among these progenitor cells control their self-renewal and differentiation, leading to the formation of glomeruli and nephron tubules from MM cells, the collecting system and ureter from UB cells, and supportive interstitial tissues from SM cells.^15–19^ Since the initiation of blood flow and urine flow takes place in embryonic kidneys during kidney development,^20^ it is possible that FSS may influence the development of embryonic kidneys. However, thus far, there has been no report on the effect of FSS on embryonic kidney cells.

While microfluidics is recognized as a useful tool in the investigation of FSS effect on kidney cells, there are limitations that impede its broad application. One of the main limitations is the use of external electro-driven pumps, such as syringe pumps and peristaltic pumps, for medium perfusion. The requirement of pumps not only limits the number of experiments that can be done simultaneously but it can also cause major complications, such as medium leakage, air bubble formation, and interfusion due to, e.g., tube connection.^21^ To solve this problem, we have previously developed a pumpless microfluidic device for tissue culture.^22^ Our pumpless device is driven by hydrostatic pressure and allows parallel experiments to be conducted simultaneously without cumbersome electronic driven equipment and intricate techniques.

In this study, using our pumpless microfluidic device, we investigated the influence of FSS on the development of one of three progenitor cell lineages in the embryonic kidneys, i.e., the ureteric bud (UB) cells. For this purpose, we have redesigned our previously reported pumpless device for tissue culture into one for cell culture experiments. We first validated the function of the redesigned device by both mathematical model and experimental measurements. With UB cells cultured in this device, we found that exposure to FSS promoted the enrichment of UB tip cells, as reflected by an increase in mRNA expression of tip cell marker genes, as well as a decrease in Dolichos Biflorus Agglutinin (DBA) binding. This represents the first report on the effect of FSS on UB cells from embryonic kidneys using pumpless microfluidic devices.

## II. MATERIALS AND METHODS

### A. Pumpless microfluidic device

**FIG. 1.**
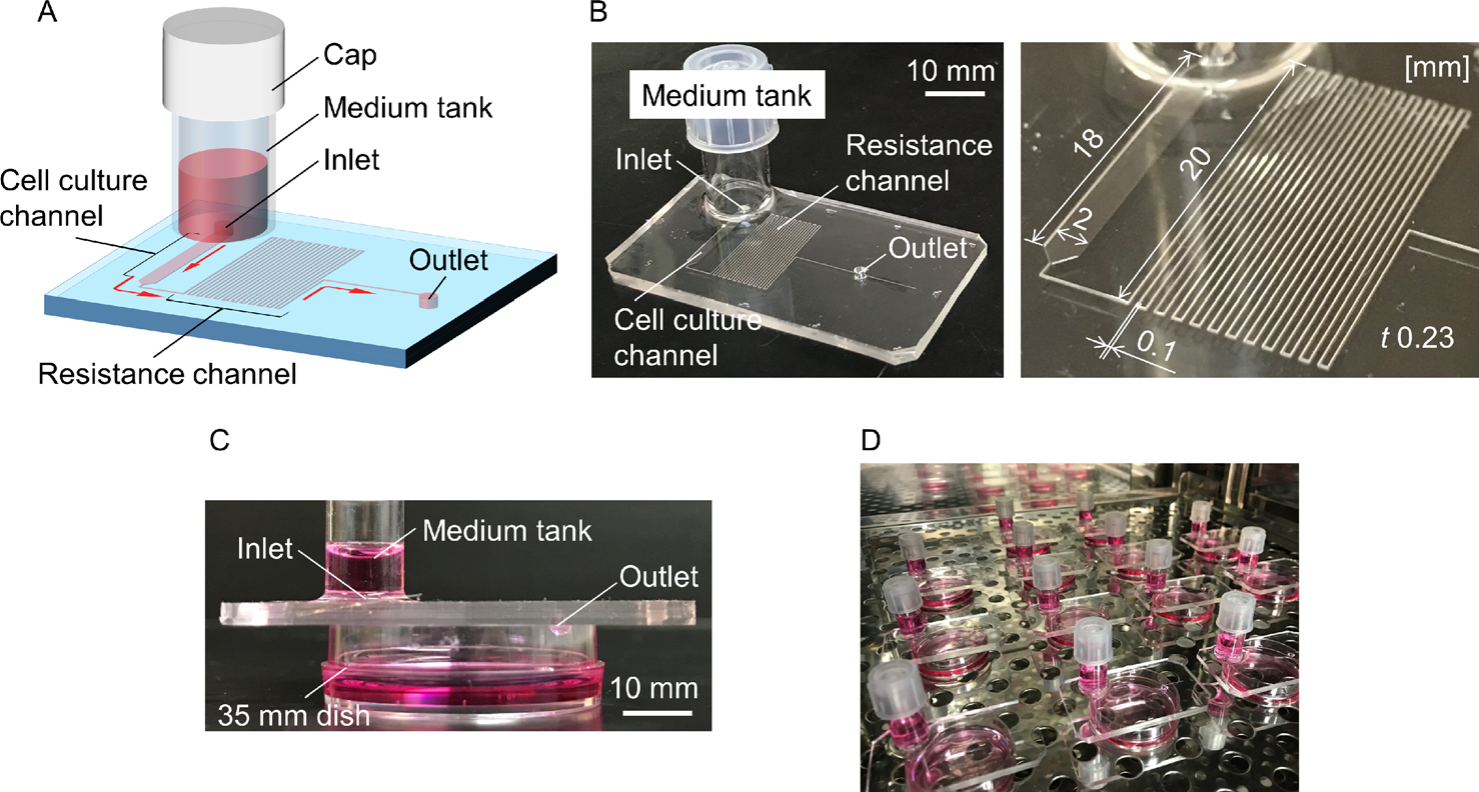
The pumpless microfluidic device. (A) Schematic 3D image of the device, showing medium tank with cap, inlet, cell culture channel, resistance channel, and outlet. (B) Photographs of the device. Overview (left) and closer view (right) of cell culture channel and resistance channel (numbers indicate scales in mm). (C) The device was put on top of a 35 mm dish, and the culture medium in the medium tank was driven by hydrostatic pressure to pass through the channels and drained into the dish through the outlet. (D) Devices in an incubator. The pumpless perfusion system of our device allowed multiple perfusion experiments to be conducted simultaneously without the need of any electronic equipment.

A pumpless device was designed based on the microfluidic device that we had previously reported.^22^ The microfluidic device consists of two parts: a medium tank and a microfluidic compartment that consists of a cell culture channel (2 mm in width, 230 *μ*m in height, and 18 mm in length) and a resistance channel (100 *μ*m in width, 230 *μ*m in height, and 500 mm in length) to regulate the medium flow velocity [Figs. 1(A) and 1(B)]. The device was placed on top of a 35 mm dish that serves as a waste pan [Fig. 1(C)]. The culture medium in the medium tank was driven by hydrostatic pressure to pass through the resistance channel before being drained into the outlet. Multiple culture experiments using this device could be run simultaneously [Fig. 1(D)].

The microfluidic device was made of dimethylpolysiloxane (PDMS) and was fabricated by conventional photolithography and soft lithography techniques.^23–25^ In brief, the SU-8 mold master was first designed and fabricated to serve as the mold to produce PDMS chips. Inlet and outlet holes on the PDMS chips were created by using a trephine. The PDMS chip with microfluidic channels and inlet hole was bonded together with another PDMS plate with outlet hole using the common oxygen plasma method. A medium tank, which was the cut half of a polystyrene tube (Falcon), was fixed to the PDMS plate with inlet hole.

### B. Primary ureteric bud cells preparation

Hoxb7^myr-Venus/+^ mice obtained from Jackson Laboratory (Bar Harbor, Main) were maintained with C57BL/6J background. Mice were genotyped using universal PCR genotyping protocols with green fluorescent protein (*GFP*) primers as shown in Table I. Embryos were genotyped under a fluorescent microscope after the isolation of kidneys. The morning of the discovery of a vaginal plug was considered as E0.5.

**TABLE I.** Primers used for qRT-PCR.

Gene		Sequence (5′-3′)
*GFP*	F	CGCACCATCTTCTTCAAGGAC
	R	TTGTGGCTGTTGTAGTTGTACTCC
*Gapdh*	F	TGAACGGATTTGGCCGTATTG
	R	ACCATGTAGTTGAGGTCAATGAAG
*Hoxb7*	F	CTTGGCGGCCGAGAGTAAC
	R	CGAGTCAGGTAGCGATTGTAGT
*Wnt11*	F	ACATGCGCTGGAACTGCT
	R	GCATACACGAAGGCTGACTC
*Ret*	F	ATGGTTGAAAACAAACTCTATGGCAT
	R	TCTTGGGAACCCAGTGCTAG
*Etv4*	F	AGCGAGTGCCCTACACCT
	R	CTGCTCATCACTGTCCGGTA
*Wnt7b*	F	TACCTAAGTTCCGCGAGGTG
	R	AGGCTTCTGGTAGCTGCGTA
*Tacstd2*	F	ACTGTACATGCCCCACCAAC
	R	GCAGGCACTTGGAAGTTAGC
*Six2*	F	CAAGTCAGCAACTGGTTCAAGA
	R	ACTGCCATTGAGCGAGGA
*Foxd1*	F	TTCGGATTCTTGGACCAGAC
	R	CAAGTCAGGGTTGCAGCATA

Hoxb7-Venus^+^ mouse embryonic kidneys were dissected free-hand from E15.5 embryos, using fine forceps under a dissecting microscope (Olympus) in Dulbecco's modified Eagle Medium high glucose (DMEM high glucose, Gibco) with 10% fetal bovine serum (FBS, Sigma). Embryonic kidneys were placed in TrypLE Express (ThermoFisher) on ice for 15 min, and then at room temperature for 10 min with shaking. The embryonic kidneys were dissociated by pipetting gently and placed in ice-cold DMEM with 20% FBS with ROCK inhibitor (Y27632, Abcam) to wash out the remaining TrypLE Express. Cell sorting was performed by fluorescence-activated cell sorting (FACS, Jazz, BD Science).

### C. Cell culture in devices

The culture medium used was DMEM high glucose supplemented with 10% FBS, 1× GlutaMAX (Gibco), 100 units penicillin ml^−1^ (Sigma), 100 *μ*g ml^−1^ streptomycin (Sigma), and 10 *μ*M Y27632 (Abcam).

All microfluidic devices were sterilized by UV irradiation for at least 1 h, followed by rinsing with phosphate-buffered saline without calcium and magnesium (PBS(-); Corning) before use. Subsequently, cell culture channels were coated with Matrigel (Corning) by filling them with Matrigel solution (Matrigel:DMEM/F12, 1:25) for 1 h at room temperature, followed by rinsing with culture medium. To inoculate UB cells into cell culture channels, cell suspension at a density of 6–8 × 10^4^ cells per device was introduced into the inlet using a micropipette. The device was held vertically to seed the cells into the channels by gravity and tapping. Cells were cultured in an incubator overnight without medium flow until the initiation of perfusion experiments. Under dynamic culture condition, continuous medium flow was initiated by adding 1.5 ml of culture medium (at 20 mm potential) into the tank every 12 h. For static culture condition, medium flow was prevented by sealing the outlet hole with a PDMS plate. The devices were placed in an incubator, where the cells were cultured in standard culture conditions (5% CO_2_, 37 °C) for 48 h. Reagents were introduced into the medium tank as indicated.

### D. Quantitative reverse transcription polymerase chain reaction (qRT-PCR)

Cultured UB cells were sampled from devices with TRIzol (ThermoFisher). The total RNA was extracted using Direct-zol RNA Microprep Kit (ZymoRearch). The extracted RNA was reverse-transcribed using iScript cDNA Synthesis Kit (BioRad) and quantified with PCR thermocycler (MyiQ cycler; BioRad) using SsoAdvanced Universal SYBR Green Supermix (BioRad). The sequences of primers used are shown in Table I.

### E. Immunocytochemistry and confocal microscopy

UB cells in cell culture channels were fixed with 4% paraformaldehyde (PFA; Thermo Scientific) in PBS for 15 min at room temperature. After washing with PBS, UB cells were treated with 0.1% TritonX-100 (Sigma) in PBS for 10 min at room temperature and then washed with PBS again. Biotinylated lectin Dolichosbiflorus agglutinin (DBA, B-1035; Vector) was then applied and incubated overnight at 4 °C. After being washed 6 times for 5 min with PBS, UB cells were incubated with PBS containing 1% bovine serum albumin (BSA) for 60 min at room temperature, followed by incubation with Avidin (NeutrAvidin, Tetramethylrhodamine conjugate, A6373; Life Technologies) for 60 min at room temperature. The nuclei were stained with DRAQ5 (Cell Signaling), diluted (1:500) in PBS, for 20 min at room temperature, and washed with PBS 6 times for 5 min at room temperature. Stained samples were examined on a laser confocal microscope (FV1000; Olympus).

### F. Statistical analysis

All values are expressed as mean ± S.D. from at least triplicate experiments. Student's t test for paired and unpaired comparison, as appropriate, was performed and differences were considered to be significant when p < 0.05.

### G. Ethics statement

This study was carried out in accordance with recommendations in the Guide for the Care and Use of Laboratory Animals of the NIH. The protocol was approved by the Institutional Animal Care and Use Committee of VA Greater Los Angeles Healthcare System (Permit Number 01002-09).

## III. RESULTS AND DISCUSSION

### A. Characterization of the pumpless microfluidic device

The flow velocity and FSS in the cell culture channel were estimated by using our previously developed mathematical model.^22^ In brief, we established a mathematical model describing the relationship between fluid potential in the medium tank and flow velocity in the resistance channel using Bernoulli's equation

$$h=\frac{v^{2}}{2g}\left(1+\lambda \frac{l}{d_{1}}\right),$$ (1)

where *h* (m) is the fluid potential in the medium tank, *v* (m s^−1^) is the flow velocity at the resistance channel, *l* (m) is the length of the resistance channel, *d*_1_ (m) is the equivalent diameter of the resistance channel, *g* (m s^−2^) is the gravitational acceleration, and *λ* is the coefficient of channel friction that can be theoretically described as *λ* = 64/*Re*, where *Re* is Reynold's number.

The flow rate, *Q* (m^3^ s^−1^), is described as follows:

$$Q=\frac{dh}{dt}A=\frac{{d_{1}}^{2}}{{d_{2}}^{2}}vA,$$ (2)

where *A* (m^2^) and *d_2_* (m) are the dimension and diameter of the medium tank, respectively. Based on Eqs. (1) and (2), both *v* and *h* can be calculated, and the flow velocity is therefore adjustable through the alteration of the length and the equivalent diameter of the resistance channel. The flow resistance of the cell culture channel is negligible because the resistance is markedly lower than that of the resistance channel.

FSS *τ* was calculated using the equation as follows:

$$\tau =\frac{6\mu Q}{ab^{2}},$$ (3)

where *μ* is the medium viscosity (g cm^−1 ^s^−1^), *Q* is the flow rate (cm^3^ s^−1^), and *a* and *b* are width (cm) and height (cm) of the cell culture channel, respectively.^7^

**FIG. 2.**
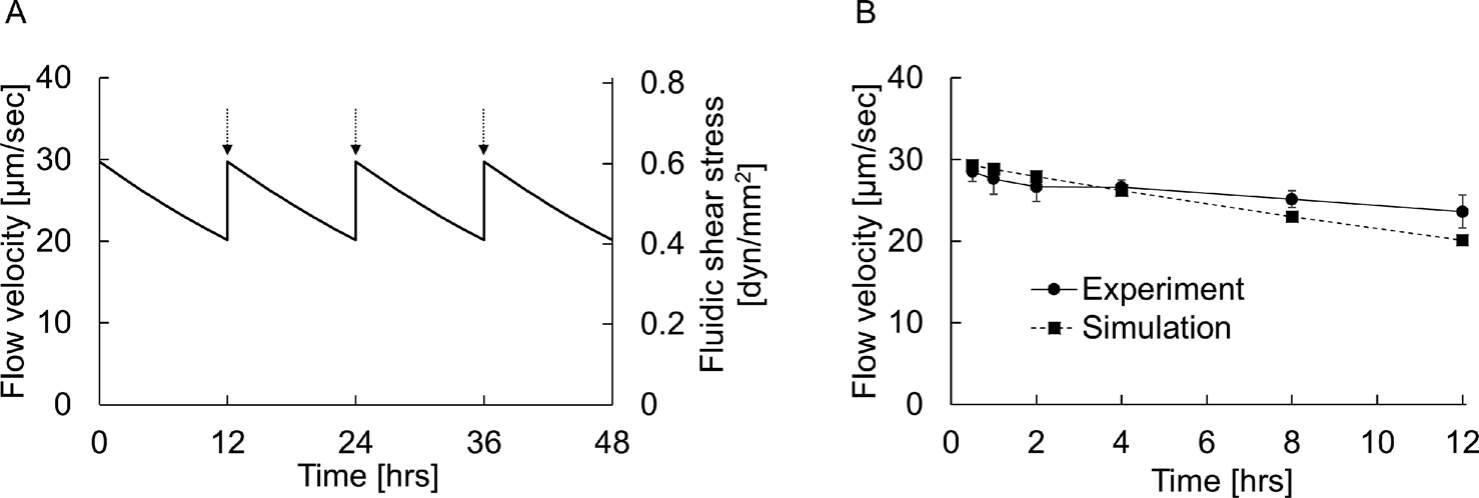
Medium flow simulation. (A) Mathematical estimation of flow velocity and FSS in cell culture channel over time for up to 48 h. Culture medium was supplied into the medium tank every 12 h as indicated by arrows. (B) Comparison of the results between simulation and experiments at 0–12 h.

We estimated the flow velocity and FSS in the cell culture chamber using our mathematical model during the culture period of up to 48 h. Culture medium was supplied into the medium tank every 12 h to keep the flow velocity constant. The FSS in our device was estimated to be in the range of 0.4–0.6 dyn mm^−2^ by the mathematical model [Fig. 2(A)]. The data showed that the channel design of our device is appropriate to realize a stable flow velocity for up to 48 h.

To further validate the function of our pumpless device, we performed experiments to measure the flow velocity and FSS levels using particle tracking velocimetry (PTV). Fluorescent microbeads of 1.0 *μ*m diameter (15702; Polysciences) diluted in culture medium (1:200) were used as tracer particles. The motion of the fluorescent beads flowing in the channel was observed using an inverted fluorescence microscope (IX73; Olympus) with a digital video camera (HDR-XR550V; Sony) at 60 fps. The velocity at the center of a cell culture channel was measured from captured images using the image processing software ImageJ (NIH), and the average velocity was calculated with a correction coefficient, which was estimated with the general purpose physics simulation software COMSOL Multiphysics (COMSOL) based on the finite element method (FEM). All measurement experiments were performed in triplicate using different devices, and the experimental data thus derived were found to match well with the simulated data using our mathematical model [Fig. 2(B)].

While the actual FSS in the developing embryonic kidneys is unknown, the range of 0.4–0.6 dyn mm^−2^ that we applied on the cultured UB cells in our study is appropriately lower than that reported in the collecting duct of the mature kidney *in vivo*.^26^ However, unlike the conventional device using a syringe pump or pressure controller, a potential disadvantage of our pumpless device is the fluctuation in the flow with a gradual decrease in flow rate due to the gradual decrease in the potential of the medium tank. Nonetheless, it is conceivable that the urine flow in the kidney tubule could be pulsatile because of the driving force of the flow being from the heart beats. Since the range and frequency of the pulsatile flow in our device are much lower than that *in vivo*,^8–10^ and the fluctuation of flow in our device could be minimized by changing the position of the medium tank and optimizing the design of microchannels based on the equation that we established, we consider that our pumpless device is suitable for our present study to evaluate the effect of FSS on UB cells *in vitro*.

### B. Effect of FSS on UB cells

**FIG. 3.**
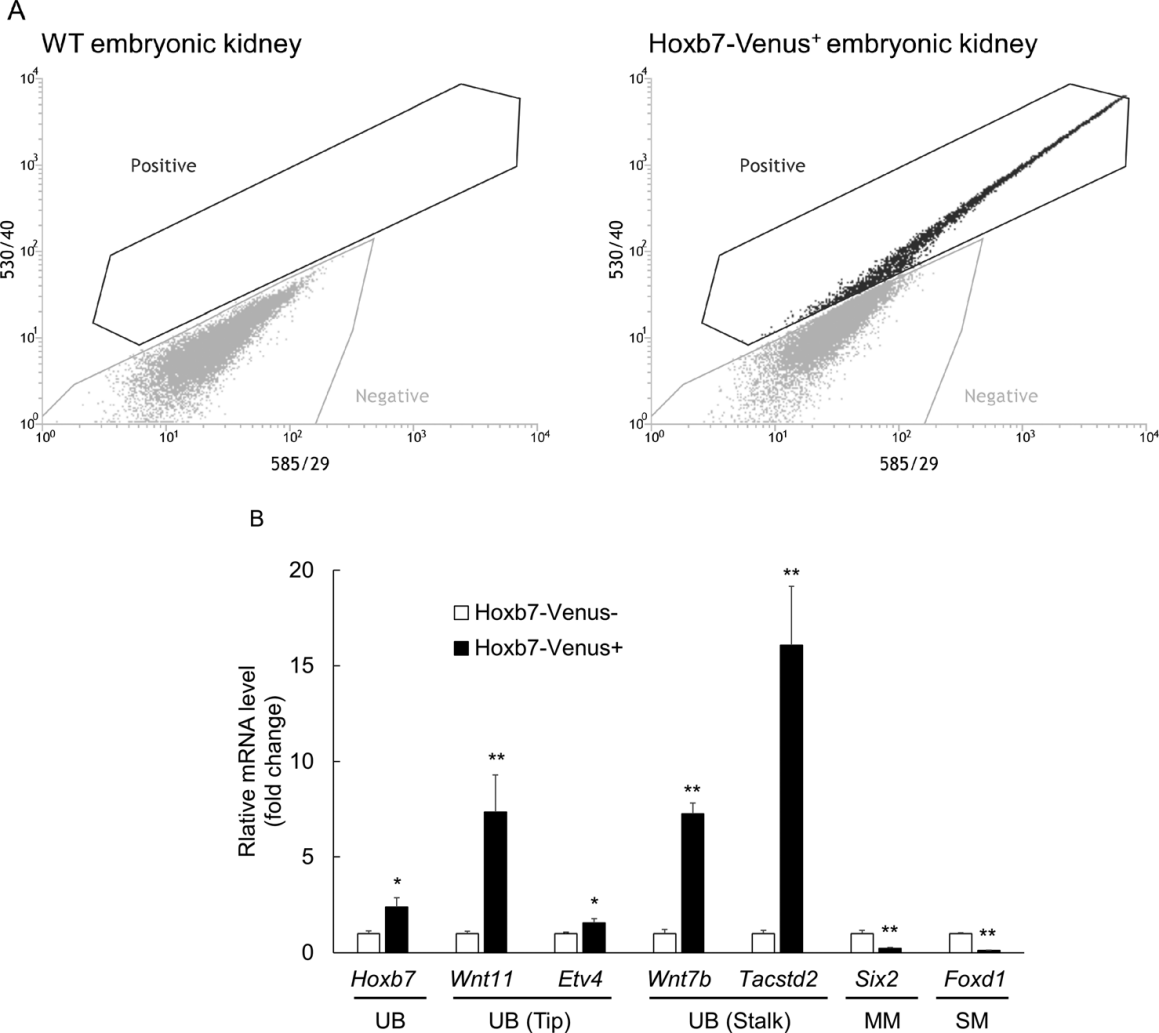
Cell sorting performed by FACS. (A) Left and right graphs show the distribution of cells dissociated from wild type (WT) and Hoxb7-Venus^+^ embryonic kidneys, respectively. The sorting gates were set by comparing the distribution of the cells between the two groups. (B) qRT-PCR results show the effective separation of UB cells from whole Hoxb7-Venus^+^ embryonic kidney cells. The mRNA expression levels of UB cell markers, such as *Hoxb7*, *Wnt11*, *Etv4*, *Wnt7b*, and *Tacstd2*, were significantly higher, while those of MM and SM cells markers, *Six2* and *Foxd1*, respectively, were significantly lower in the Hoxb7-Venus^+^ population, as compared to Hoxb7-Venus^−^ population. Data were normalized with *Gapdh* expression level (n = 4; *p < 0.05; and **p < 0.01 versus Hoxb7-Venus^−^).

In all experiments where UB cells were purified by FACS, we set the sorting gates for negative and positive populations by comparing samples derived from wild-type and Hoxb7-Venus^+^ embryonic kidneys as shown in Fig. 3(A). The purification of the UB cells was further verified by the expression of predominantly UB markers, such as *Hoxb7*, *Wnt11*, *Etv4*, *Wnt7b*, and *Tacstd2*, with minimal detection of markers for the remaining two lineages of cells in the embryonic kidneys, i.e., the metanephric mesenchymal (MM) cell marker, *Six2*, and stromal (SM) cell marker, *Foxd1* [Fig. 3(B)].

**FIG. 4.**
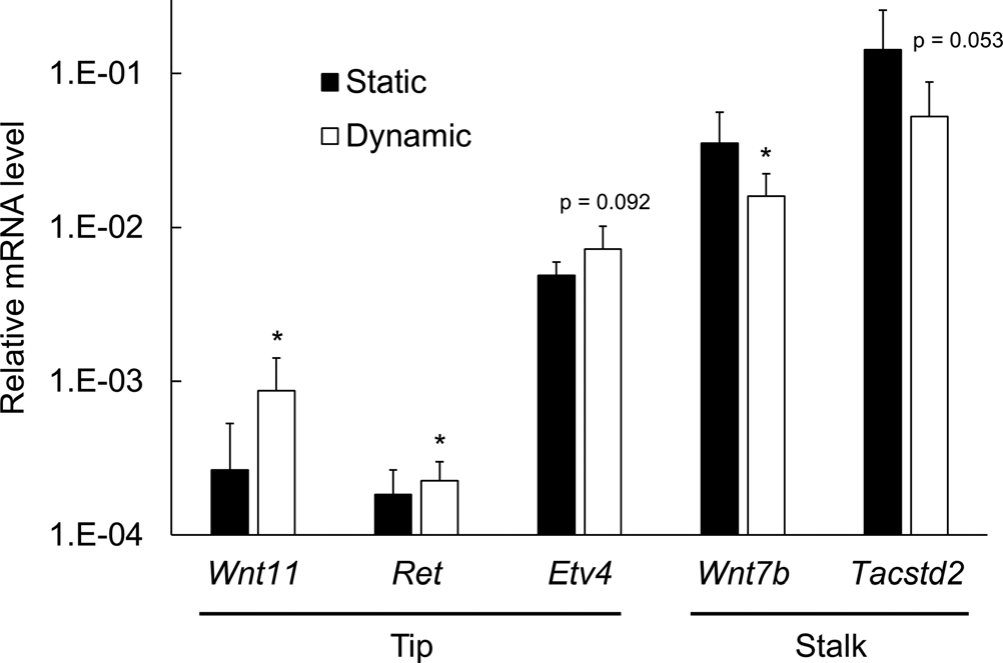
Effect of fluidic shear stress on the mRNA expression levels of UB tip vs. stalk marker genes. qRT-PCR results show that, as compared to samples under static culture condition (Static), exposure to fluidic shear stress for 48 h (Dynamic) led to a significant increase in the mRNA expression levels of tip marker genes, including *Wnt11* and *Ret*, and a significant decrease in that of stalk marker gene, *Wnt7b*. Despite not reaching statistical significance, there was also a similar trend of change in another tip marker gene *Etv4* (p = 0.092) and stalk marker gene *Tacstd2* (p = 0.053). Data were normalized by *Gapdh* expression levels. (n = 5 independent replicates; *p < 0.05 versus static condition).

As shown in Fig. 4, we found that, as compared to UB cells under static culture condition, exposure of UB cells to FSS for 48 h led to a significant increase in the mRNA expression levels of UB tip cell marker genes, including *Wnt11* and *Ret*, together with a significant decrease in UB stalk cell marker gene, *Wnt7b*. There was also a similar trend of changes in another tip marker gene *Etv4* (p = 0.092) and stalk marker gene *Tacstd2* (p = 0.053), although they did not reach statistical significance.

**FIG. 5.**
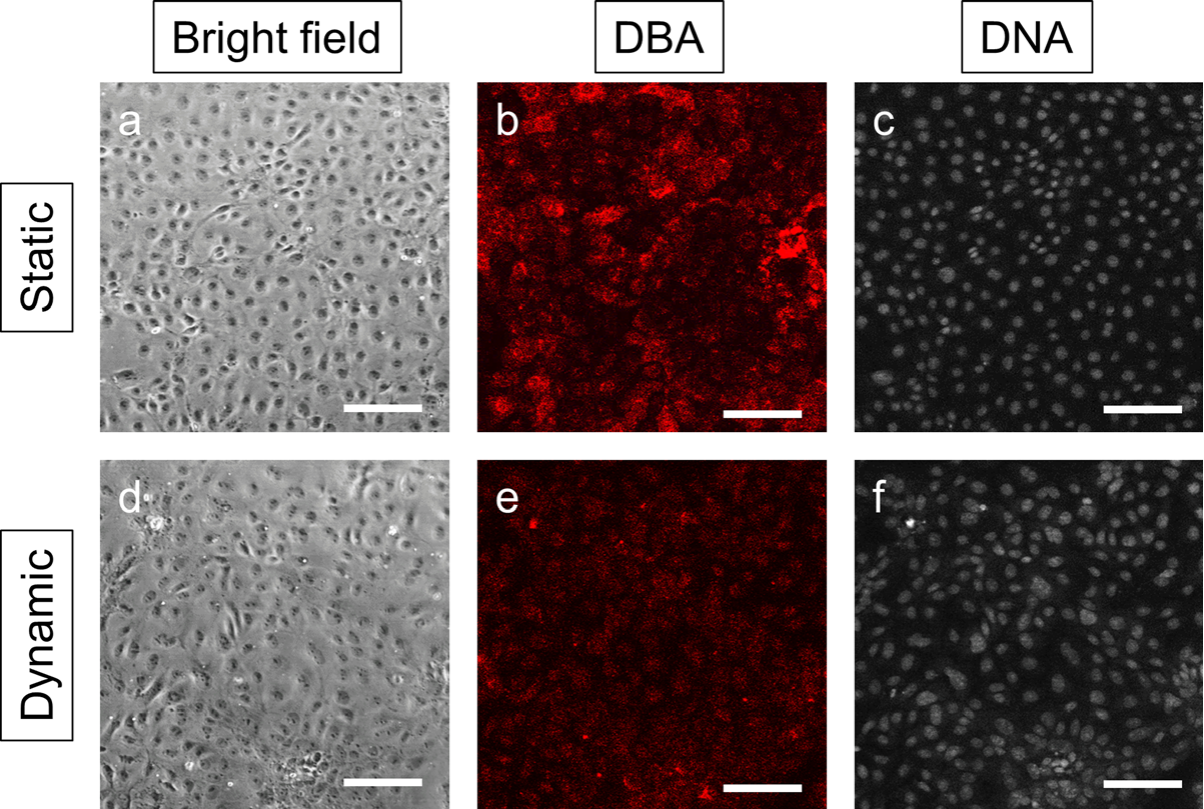
Effect of FSS on UB cell morphology and DBA binding. There was no noticeable change in UB cell morphology under bright field microscopy after culture in either static (a) or dynamic (d) conditions for 48 h. Consistent with qRT-PCR results, there was a remarkable decrease in DBA binding in UB cells under dynamic culture condition as compared to static culture condition (b vs. e), suggesting an increase in UB tip cells after exposure to FSS for 48 h (Scale bars, 100 *μ*m).

To lend further support for the enrichment of UB tip cells under dynamic culture condition, we also found that the exposure to FSS for 48 h led to a remarkable reduction in the binding of lectin DBA (Fig. 5), which is a well described characteristic for UB tip cells as compared to UB stalk cells.^27^ There was no noticeable morphology change in UB cells under bright field microscopy.

Put together, we conclude that the exposure to FSS on the *in vitro* cultured UB cells led to an enrichment in tip cell population. The mechanism underlying such effect of FSS is not immediately clear from our study. It is possible that FSS may preferentially stimulate the proliferation of tip cells over stalk cells. It is also possible that stalk cells are more vulnerable to the cell injury effect of FSS. On the other hand, in view of the well demonstrated plasticity between UB tip and stalk cells, where the transition between these two types of UB cells can take place in a reversible fashion,^28^ it is also conceivable that FSS may preferentially induce the transition of stalk cells to become tip cells.

The cellular signaling pathway(s) that mediate the effect of FSS to enrich tip cell population is not clear. Various factors and signaling pathways have been shown to regulate UB outgrowth and branching morphogenesis,^17,29,30^ such as Wnt-β-catenin signaling, fibroblast growth factor (FGF) and bone morphogenetic protein (BMP) signaling, retinoic acid signaling, glial-cell-derived neurotrophic factor (GDNF)-Ret signaling, mTOR, and Hippo signaling pathways.^31,32^ It is possible that FSS could have triggered these signaling pathways in UB cells and led to the enrichment of tip cell population. In this regard, it is of interest to note that FSS has been shown to trigger Wnt/β-catenin signaling in lymphatic endothelial cells,^33^ while Wnt/β-catenin signaling is known to be essential in maintaining UB cells in a precursor state.^34^ FSS has also been shown to activate mTOR-dependent pathway in kidney tubules,^35^ while mTOR pathway is known to maintain *Ret* expression in UB tip cells.^31^ Further studies are required to clarify these possibilities and identify the signaling pathway(s) that mediates the effect of FSS in UB cells.

Irrespective of the underlying mechanism, it is generally thought that in embryonic kidneys, urine flow initiates around E15.5 when blood flow and glomerular filtration take place,^20^ and since UB tip cells are known to be the proliferating progenitor cells that contribute to branching morphogenesis and development of the collecting system in the kidney,^29^ our finding that FSS led to an enrichment of tip cell population in the *in vitro* cultured UB cells derived from E15.5 embryonic kidneys could have an important implication for the development and function of the kidney.

## IV. CONCLUSION

In this study, we evaluated the influence of FSS on *in vitro* cultured UB cells using a pumpless microfluidic device. We found from our present study that exposure to FSS led to an increase in mRNA expression levels of UB tip cell marker genes with a decrease in stalk cell marker genes. We also found that exposure to FSS led to a remarkable reduction in the binding of lectin Dolichos Biflorus Agglutinin (DBA), characteristic of UB tip cells. In conclusion, results of our present study show that exposure to FSS led to an enrichment in UB tip cell population, which could imply an important link between the FSS from the initiation of urine flow and the development and function of the kidney. As shown in this study, novel engineering technologies, such as microfluidics, may help to introduce a new paradigm for biological science. Our pumpless devices, which allow parallel cell culture experiments without cumbersome electronic driven equipment and intricate techniques, could further facilitate collaborative studies among researchers from different fields.

## References

[1] E. M. Frohlich , X. Zhang , and J. L. Charest , Integr. Biol. 4(1), 75–83 (2012). 10.1039/C1IB00096A 22139064

[2] S. M. Nauli , F. J. Alenghat , Y. Luo , E. Williams , P. Vassilev , X. Li , A. E. H. Elia , W. Lu , E. M. Brown , S. J. Quinn , D. E. Ingber , and J. Zhou , Nat. Genet. 33, 129 (2003). 10.1038/ng1076 12514735

[3] R. Carrisoza-Gaytan , Y. Liu , D. Flores , C. Else , H. G. Lee , G. Rhodes , R. M. Sandoval , T. R. Kleyman , F. Y. Lee , B. Molitoris , L. M. Satlin , and R. Rohatgi , Am. J. Physiol.: Renal Physiol. 307(2), F195–F204 (2014). 10.1152/ajprenal.00634.2013 24872319 PMC4152160

[4] R. D. Sochol , N. R. Gupta , and J. V. Bonventre , Curr. Transplant. Rep. 3(1), 82–92 (2016). 10.1007/s40472-016-0085-x 28090431 PMC5232415

[5] H.-C. Huang , Y.-J. Chang , W.-C. Chen , H. I. C. Harn , M.-J. Tang , and C.-C. Wu , Tissue Eng., Part A 19(17–18), 2024–2034 (2013). 10.1089/ten.tea.2012.0605 23557379 PMC3726225

[6] M. Zhou , H. Ma , H. Lin , and J. Qin , Biomaterials 35(5), 1390–1401 (2014). 10.1016/j.biomaterials.2013.10.070 24239111

[7] K. J. Jang , A. P. Mehr , G. A. Hamilton , L. A. McPartlin , S. Chung , K. Y. Suh , and D. E. Ingber , Integr. Biol. 5(9), 1119–1129 (2013). 10.1039/c3ib40049b 23644926

[8] Y. Duan , N. Gotoh , Q. Yan , Z. Du , A. M. Weinstein , T. Wang , and S. Weinbaum , Proc. Natl. Acad. Sci. U. S. A. 105(32), 11418–11423 (2008). 10.1073/pnas.0804954105 18685100 PMC2516248

[9] N. Ferrell , R. R. Desai , A. J. Fleischman , S. Roy , H. D. Humes , and W. H. Fissell , Biotechnol. Bioeng. 107(4), 707–716 (2010). 10.1002/bit.22835 20552673 PMC3903011

[10] D. Maggiorani , R. Dissard , M. Belloy , J. S. Saulnier-Blache , A. Casemayou , L. Ducasse , S. Gres , J. Belliere , C. Caubet , J. L. Bascands , J. P. Schanstra , and B. Buffin-Meyer , PLoS One 10(7), e0131416 (2015). 10.1371/journal.pone.0131416 26146837 PMC4493045

[11] N. A. Stathopoulos and J. D. Hellums , Biotechnol. Bioeng. 27(7), 1021–1026 (1985). 10.1002/bit.260270713 18553772

[12] M. P. Hoenig and M. L. Zeidel , Clin. J. Am. Soc. Nephrol. 9(7), 1272–1281 (2014). 10.2215/CJN.08860813 24789550 PMC4078957

[13] M. H. Little and A. P. McMahon , Csh Perspect Biol. 4(5), 1–18 (2012). 10.1101/cshperspect.a008300 PMC3331696 22550230

[14] F. Costantini and R. Kopan , Dev. Cell 18(5), 698–712 (2010). 10.1016/j.devcel.2010.04.008 20493806 PMC2883254

[15] G. R. Dressler , Development (Cambridge, England) 136(23), 3863–3874 (2009). 10.1242/dev.034876 PMC2778737 19906853

[16] Q. C. Vega , C. A. Worby , M. S. Lechner , J. E. Dixon , and G. R. Dressler , Proc. Natl. Acad. Sci. U. S. A. 93(20), 10657–10661 (1996). 10.1073/pnas.93.20.10657 8855235 PMC38210

[17] V. K. Nagalakshmi and J. Yu , Mol. Reprod. Dev. 82(3), 151–166 (2015). 10.1002/mrd.22462 25783232 PMC4376585

[18] C. J. Rowan , S. Sheybani-Deloui , and N. D. Rosenblum , Results Probl. Cell Differ. 60, 205–229 (2017). 10.1007/978-3-319-51436-9 28409347

[19] D. Fanni , C. Gerosa , L. Vinci , R. Ambu , A. Dessi , P. Van Eyken , V. Fanos , and G. Faa , J. Matern-Fetal Neonat. Med. 29(23), 3815–3820 (2016). 10.3109/14767058.2016.1147553 26866875

[20] C. Rymer , J. Paredes , K. Halt , C. Schaefer , J. Wiersch , G. Zhang , D. Potoka , S. Vainio , G. K. Gittes , C. M. Bates , and S. Sims-Lucas , Am. J. Physiol.: Renal Physiol. 307(3), F337–F345 (2014). 10.1152/ajprenal.00208.2014 24920757 PMC4121567

[21] Y. Wang , D. Lee , L. Zhang , H. Jeon , J. E. Mendoza-Elias , T. A. Harvat , S. Z. Hassan , A. Zhou , D. T. Eddington , and J. Oberholzer , Biomed. Microdevices 14(2), 419–426 (2012). 10.1007/s10544-011-9618-3 22252566 PMC3303988

[22] M. Komeya , K. Hayashi , H. Nakamura , H. Yamanaka , H. Sanjo , K. Kojima , T. Sato , M. Yao , H. Kimura , T. Fujii , and T. Ogawa , Sci. Rep. 7(1), 15459 (2017). 10.1038/s41598-017-15799-3 29133858 PMC5684205

[23] T. Fujii , Microelectron. Eng. 61–62, 907–914 (2002). 10.1016/S0167-9317(02)00494-X

[24] H. Kimura , T. Yamamoto , H. Sakai , Y. Sakai , and T. Fujii , Lab Chip 8(5), 741–746 (2008). 10.1039/b717091b 18432344

[25] M. Horayama , K. Shinha , K. Kabayama , T. Fujii , and H. Kimura , PLoS One 11(12), e0168158 (2016). 10.1371/journal.pone.0168158 27930750 PMC5145238

[26] W. Liu , N. S. Murcia , Y. Duan , S. Weinbaum , B. K. Yoder , E. Schwiebert , and L. M. Satlin , Am. J. Physiol.: Renal Physiol. 289(5), F978–F988 (2005). 10.1152/ajprenal.00260.2004 15972389

[27] L. Michael , D. E. Sweeney , and J. A. Davies , J. Anat. 210(1), 89–97 (2007). 10.1111/j.1469-7580.2006.00670.x 17229286 PMC2100263

[28] D. Sweeney , N. Lindstrom , and J. A. Davies , Development (Cambridge, England) 135(15), 2505–2510 (2008). 10.1242/dev.022145 18579677

[29] F. Costantini , Wiley Interdiscip. Rev.: Dev. Biol. 1(5), 693–713 (2012). 10.1002/wdev.52 22942910 PMC3430146

[30] S. Yuri , M. Nishikawa , N. Yanagawa , O. D. Jo , and N. Yanagawa , Stem Cell Rep. 8(2), 401–416 (2017). 10.1016/j.stemcr.2016.12.011 PMC5311471 28089670

[31] N. Kojima , H. Saito , M. Nishikawa , S. Yuri , O. D. Jo , P. C. Pham , N. Yanagawa , and N. Yanagawa , Cell. Signalling 23(2), 371–379 (2011). 10.1016/j.cellsig.2010.10.007 20940044

[32] A. Reginensi , L. Enderle , A. Gregorieff , R. L. Johnson , J. L. Wrana , and H. McNeill , Nat. Commun. 7, 12309 (2016). 10.1038/ncomms12309 27480037 PMC4974664

[33] B. Cha , X. Geng , M. R. Mahamud , J. X. Fu , A. Mukherjee , Y. Kim , E. H. Jho , T. H. Kim , M. L. Kahn , L. J. Xia , J. B. Dixon , H. Chen , and R. S. Srinivasan , Genes Dev. 30(12), 1454–1469 (2016). 10.1101/gad.282400.116 27313318 PMC4926867

[34] T. D. Marose , C. E. Merkel , A. P. McMahon , and T. J. Carroll , Dev. Biol. 314(1), 112–126 (2008). 10.1016/j.ydbio.2007.11.016 18177851 PMC2699621

[35] K. R. Long , K. E. Shipman , Y. Rbaibi , E. V. Menshikova , V. B. Ritov , M. L. Eshbach , Y. Jiang , E. K. Jackson , C. J. Baty , and O. A. Weisz , Mol. Biol. Cell 28(19), 2508–2517 (2017). 10.1091/mbc.e17-04-0211 28720662 PMC5597323

